# Dietary Habits, Fatty Acids and Carotenoid Levels Are Associated with Neovascular Age-Related Macular Degeneration in Chinese

**DOI:** 10.3390/nu11081720

**Published:** 2019-07-25

**Authors:** Alex L.-K. Ng, Ho Hang Leung, Ryo Kawasaki, Wing-Lau Ho, Loraine L.-W. Chow, Sharon S.-W. Chow, Jetty Chung-Yung Lee, Ian Y.-H. Wong

**Affiliations:** 1 Department of Ophthalmology, LKS Faculty of Medicine, The University of Hong Kong, Sassoon Road, Hong Kong; 2 Hong Kong Ophthalmic Associate, Queen’s Road, Hong Kong; 3 School of Biological Sciences, The University of Hong Kong, Pokfulam Road, Hong Kong; 4 Department of Vision Informatics, Osaka University, 2-2 Yamadaoka, 565-0871 Osaka, Japan; 5 Grantham Hospital, Hospital Authority, Wong Chuk Hang, Hong Kong; 6 Department of Ophthalmology, Hong Kong Sanatorium and Hospital, Happy Valley, Hong Kong

**Keywords:** macular degeneration, polyunsaturated fatty acid, saturated fatty acid, carotenoids

## Abstract

The role of diet and circulatory carotenoids and docosahexaenoic acid (DHA) and eicosapentaenoic acid (EPA) are implicated in age-related macular degeneration (AMD) but not well studied in Chinese. However, other fatty acids were not comprehensively evaluated if it had additional consequence on AMD. This study investigated the relationship among dietary habits, fatty acids levels, carotenoids and AMD in Hong Kong Chinese adults. In this cross-sectional case-controlled study, plasma fatty acids including, saturated fatty acids (SFA), monounsaturated fatty acids (MUFA) and polyunsaturated fatty acids (PUFA), and carotenoids levels were quantified between patients with neovascular AMD (*n* = 99) and age-gender-matched controls (*n* = 198). A food frequency questionnaire was also conducted. Low blood carotenoid levels and omega-3 PUFAs namely DHA, EPA and α-linolenic acid increased the odds ratio of developing neovascular AMD. High blood omega-6 PUFAs specifically arachidonic acid and eicosadienoic acid, oleic acid (a MUFA) and SFA levels increased the odds ratio of having neovascular AMD. Neovascular AMD group had significantly less omega-3 PUFA rich food (vegetables, nuts, seafood) intake and higher SFA (meat) intake than controls. In short, neovascular AMD was associated with lower circulatory levels of carotenoids and omega-3 PUFAs, and higher level of omega-6 PUFAs, oleic acid and SFAs in the Hong Kong Chinese population. These findings enhance the understandings of dietary impacts on neovascular AMD and provide a context for future nutritional intervention studies.

## 1. Introduction

Age-related macular degeneration (AMD) is a leading cause of blindness in the developed world and mainly affects people over 50 years of age. The role of specific dietary nutrients, such as, polyunsaturated fatty acids (PUFA), and carotenoids (lutein, zeaxanthin and beta-carotene) plus vitamins C and E, zinc and copper are effective in reducing the risk of development and progression from preclinical AMD to a more advanced form of AMD owing to their antioxidant and photo-protective properties [1]. However, unlike the current study, these investigations did not isolate the effect of the carotenoids or showed a prospective protection by PUFAs [2,3].

The most important PUFA in humans are the omega-3 PUFA, which include α-linolenic acid (ALA), eicosapentaenoic acid (EPA) and docosahexaenoic acid (DHA). ALA is a precursor of EPA, which is a precursor of DHA in the omega-3 PUFA metabolism. DHA is an important primary structural component of the retina. Humans cannot synthesize PUFA, therefore, marine fish in particular, is the main source of EPA and DHA. EPA and DHA have anti-inflammatory properties, and a reduced level is implicated in AMD [4]. A diet rich in meat and dairy has high omega-6 PUFA levels and in contrast to omega-3 PUFA, these are sources of pro-inflammatory eicosanoids and have competitive interactions with omega-3 PUFAs in the metabolism [4]. In addition, carotenoids (i.e., lutein, zeaxanthin and beta-carotene) are important antioxidants, and being fat-soluble, they interact with PUFA metabolism. Moreover, lutein and zeaxanthin are the main components of the macular pigment.

Increased dietary intake of low-glycemic index foods, omega-3 PUFA and lutein/zeaxanthin is considered useful in the prevention of AMD or in retarding its progression [1,5,6]. A number of large-scale trials have investigated the effects of nutritional supplements in preventing disease progression. The Age-Related Eye Disease Study (AREDS) in particular, has resulted in the development of evidence-based supplements for preventing such progression [2]. Subsequently, several randomized prospective studies have shown beneficial effects of omega-3 PUFA, lutein and zeaxanthin in reducing the development of AMD and increasing macular pigment optical density [3,7,8,9,10,11,12]. In the landmark AREDS2 study, although there was no additional benefit of adding DHA and EPA on top of supplements originally proposed in the AREDS, the subgroup with low lutein and zeaxanthin level had the biggest benefit from these supplements [3].

Most studies to date have focused on the effects of antioxidants and omega-3 PUFA supplements. There are very few studies looking at the relationship of circulatory levels of fatty acids and carotenoids, and AMD, especially in the Asian population where dietary intake of lipids is different compared to Western populations. We hypothesized that the AMD patients will have a worse PUFA profile (lower omega-3 PUFA and higher omega-6 PUFA) and lower antioxidant level (carotenoids, including lutein, zeaxanthin and beta-carotene) and that there will be a close association between those levels and dietary habits. The aim of the current study is to study the relationship between the blood levels of multiple fatty acids and antioxidants (carotenoids), dietary habits and AMD in our local population.

## 2. Materials and Methods

### 2.1. Study Design

This is a cross-sectional case-controlled study performed at the Queen Mary Hospital and Grantham Hospital, Hong Kong over a 2-year period from December 2015 to December 2017. The blood PUFA profile and antioxidant levels between patients with exudative AMD and age-gender-matched controls were determined. The study was approved by the Institutional Review Board of the University of Hong Kong/Hospital Authority Hong Kong West Cluster (Ref number: UW 14-405) and adhered to the tenets of the Declaration of Helsinki. All patients had signed an informed consent before joining the study.

Exudative AMD was defined by usual clinical features with evidence of choroidal neovascularization leakage on fundus fluorescein angiography and indocyanine green angiography and presence of subretinal fluid on optical coherence tomography. Only patients aged 50 or older were recruited. For the control group, patients who matched the subjects in age were invited to act as control subjects, if they did not display any clinical evidence of AMD, defined by absence of drusen > 63 micrometers or pigmentary abnormalities [2]. Exclusion criteria of both AMD group and control group included: Age less than 50; non-Chinese ethnicity; myopia more than -6D; presence of any other retinal vascular disease (defined as presence of microaneurysm or blot retinal hemorrhages); history of any previous vitrectomy surgeries; history of cataract surgery or other intraocular surgeries within 6 months; history of uveitis; on systemic medication that would cause pigmentary maculopathy: hydroxychloroquine, chloroquine, thioridazine, deferoxamine; and those unable or unwilling to provide consent. The recruitment of exudative AMD subjects and controls were conducted within the same study period from the retinal clinical and general ophthalmology clinic of the study sites. The blood taking was performed by the recruiting ophthalmologists and the questionnaire (Supplementary Material 1) was performed by a research assistant. The blood samples were then analyzed by the School of Biological Sciences, The University of Hong Kong.

### 2.2. Calculation of Sample Size

Prior to the main study, the sample size was calculated based on our pilot data with 21 AMD patients and 25 age-matched controls; it was planned to determine a continuous response variable from independent control and experimental subjects with 2 control(s) per experimental subject. In a previous study the response within each subject group was normally distributed with standard deviation 0.5 [13]. Assuming the true difference in the experimental and control means was 0.2, we needed to study 99 experimental subjects and 198 control subjects (regardless of the age) to be able to reject the null hypothesis that the population means of the experimental and control groups are equal with probability (power) 0.9. The Type I error probability associated with this test of this null hypothesis is 0.05.

### 2.3. Measurement of Plasma Fatty Acids and Carotenoids

Plasma fatty acids were extracted and quantified using gas chromatography-mass spectrometry method as described by Quehenberger et al. with modification [14]. The list of fatty acids analyzed is summarized in Supplementary Material 2. Carotenoids (beta-carotene, lycopene and lutein + zeaxanthin) in the plasma samples were extracted and quantified using liquid chromatography with diode array detector method as described by Karppi et al. with modification [15]. The detailed steps of the plasma fatty acids analysis and carotenoids analysis are described in Supplementary Material 2.

### 2.4. Food Frequency Questionnaire

Questionnaire data were also collected from all recruited subjects. The questionnaire used in the study was modified from the ‘‘Food Frequency Questionnaire” compiled by the School of Public Health that was validated for Hong Kong Chinese [16,17]. The aim of the questionnaire was to collect information on dietary habits. It is specifically catered for Hong Kong Chinese and consists of detailed question on the type of food and the method of preparation. The questionnaire (attached as Supplementary Material 1) is very comprehensive so as to reflect the patient’s diet as close as possible and to avoid biased opinion (i.e., if we only target 1–2 food items, patient will know these are related to their disease). Both Chinese and English versions were available. The questionnaire also collects information on subject demographics and BMI. A trained research assistant who is blinded to the group allocation administered the questionnaire.

### 2.5. Statistical Analysis

The levels of each fatty acid and carotenoid in the AMD and control groups were compared using an independent t-test (parametric) or Mann-Whitney-U test (non-parametric). The mean difference of the two groups was then adjusted for age and sex. The dietary habits of the two groups, based on their questionnaire scores were also be compared using an independent t-test or Mann-Whitney-U test for continuous scores or chi-square test for categorical variables. Conditional logistic regression model was used to adjust for the multiple variables to calculate the odds ratios (ORs) of having AMD and the level of each fatty acid and carotenoid (lutein, zeaxanthin and beta-carotene) in the blood between top and bottom quartile. We then applied the Principal Component Analysis (PCA) to summarize multiple fatty acids into 8 groups of high correlation. We then used the logistic regression analysis using the principal component (PC) 1 to PC 8 to see which component is associated with being AMD cases. All analysis was performed by SPSS (v. 21, IBM Corp., Armonk, NY, USA).

## 3. Results

A total of 99 (62 males and 37 females) exudative AMD patients and 198 (97 males and 101 females) age-matched controls were recruited in the study period. The mean age was 73.7 ± 10.2 years old in AMD and 67.1 ± 9.3 years old in control. There was no significant difference in BMI between controls (24.48 ± 3.81 kg/m^2^) and AMD (24.17 ± 3.22 kg/m^2^). The mean difference of all tested carotenoids and fatty acids between AMD cases and controls after adjustment for age and sex are shown in Table 1.

**Figure 1:**
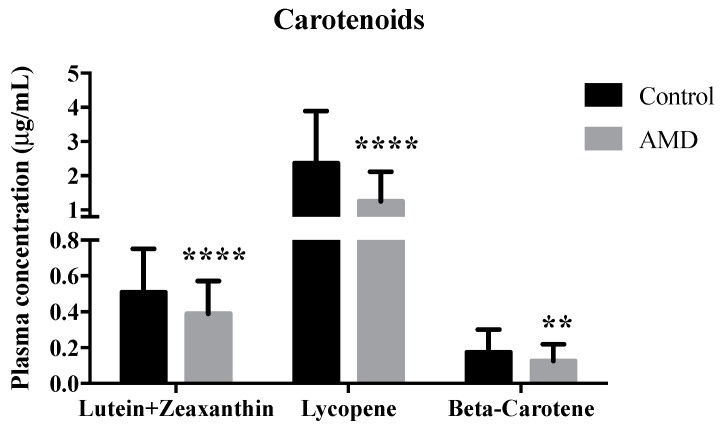
Plasma concentration of carotenoids in healthy control and patients with wet age-related macular degeneration (AMD). Values are expressed as mean ± SD. ** *p* < 0.01 and **** *p* < 0.0001 versus healthy control.

Of all tested carotenoids, including lutein and zeaxanthin combined, beta-carotene and lycopene, they were significantly lower in AMD subjects compared to controls (*p* = 0.013, 0.036 and 0.001 respectively) (Figure 1). For omega-3 PUFAs, EPA and DHA were significantly lower in AMD subjects than the control group (*p* < 0.0005 and *p* = 0.002 respectively) (Table 2). In contrary, the eicosatrienoic acid was significantly higher in AMD group. Other omega-3 PUFAs including ALA and docosapentaenoic acid were inconsistently lower in AMD subjects (0.05 < *p* < 0.1). For omega-6 PUFAs, arachidonic acid (ARA) level (*p* < 0.0005) and eicosadienoic acid level (*p* = 0.004) were significantly higher in AMD subjects, but dihomo-γ-linolenic acid was lower (*p* = 0.002). For the monounsaturated fatty acids (MUFAs), the oleic acid level and heptadecenoic acid level were significantly higher in AMD group. Most saturated fatty acids (SFAs) were significantly higher in AMD group, except for stearic acid and arachidic acid (Table 2).

We analyzed the blood level of all tested carotenoids and fatty acids and divided the levels into four quartiles. Using the highest quartile as a reference, we calculated these subjects with blood levels at the lowest quartile for the odds ratio in developing AMD. Those with a statistically significant results are summarized in Table 3. When the subjects had low carotenoid levels (when comparing the bottom quartile to top quartile), the odds ratio of having AMD was 2.78 for beta-carotene, 6.02 for lycopene, and 4.82 for combined lutein and zeathanxin. A low level of omega-3 PUFA increased the odds ratio of developing AMD by 6.33 for DHA, 7.79 for EPA and 3.11 for ALA. In contrary, a low level of ARA and eicosatrienoic acid were protective, with odds ratio being 0.11 and 0.16 respectively.

We then used the lowest quartile as a reference and calculated the subjects’ blood fatty acid levels at the highest quartile for the odds ratio in developing AMD. Those with statistically significant results are summarized in Table 4. When the subjects had high levels of eicosadienoic acid (omega-6 PUFA), oleic acid (MUFA), palmitic acid (SFA) and pentadecylic acid (SFA), when comparing the top quartile to bottom quartile, the odds ratio of having AMD were 2.63, 14.10, 11.48 and 33.75 respectively.

Using the PCA, we summarized multiple fatty acids into eight PC groups of high correlation. Each had different representations of multiple fatty acids. We then applied a logistic regression analysis using the PC 1 to PC 8 to understand which component was associated to AMD cases. We found that the PC 1, consisting of C15:0 (pentadecylic acid), C16:0 (palmitic acid), C17:0 (margaric acid), C18:1n9 (oleic acid) and C20:3n3 (eicosatrienoic acid), was significantly associated with developing AMD, while PC 3, consisting of C18:0 (stearic acid), C20:2n6 (eicosadienoic acid) and C20:3n6 (dihomo-γ-linolenic acid) as well as PC 5, which consisted of C16:1n9 (palmitoleic acid), C20:5n3 (EPA) and C22:6n3 (DHA), were significantly protective.

For the questionnaire, a statistically significant difference was found in the median score and interquartile range between the two groups in eight items (Table 5): AMD group had higher intake frequency in Chinese preserved vegetable (*p* = 0.045) and preserved meats (*p* = 0.025). For vegetables, the AMD group had a lower serving size of both green leafy vegetables (Choi Sum, Pok Choi, Chinese kale, broccoli, spinach) (*p* < 0.001) and other vegetables (turnip, celery cabbage, cabbage and potatoes) (*p* < 0.001) when compared with control. The AMD group also had higher frequency in eating red meat including pork, beef and lamb (*p* = 0.012) and poultry (*p* = 0.031) compared with controls, although controls had higher serving size in poultry each time (*p* = 0.030). For seafood, fish intake did not show significant difference between both groups, but AMD group had significantly less frequency of intake in other seafood (prawn, shrimp, crab, mussels, scallop) than in controls (*p* = 0.006).

## 4. Discussion

Population-based studies indicate that AMD is more prevalent in Caucasians than in Asian origin people [18,19,20]. However, in Asian populations, the more severe form of AMD i.e., exudative (or wet) type is prevalent [21,22]. Some investigators have hypothesized that the lower incidence of the disease in Japan may be related to higher antioxidant content of the typical Japanese diet [20]. In a recent study in Hong Kong, the age-standardized prevalence of early AMD was 17.9% and late AMD was 0.1% [23]. Most studies to date have focused on the effects of supplements, and there are very few studies looking at the relationship of the circulatory levels of these nutrients and AMD. Among the limited data available, a few Western studies did show a lowered risk of AMD with a high combination of EPA plus DHA levels in the diet or in circulation [7,13]. Also, most of the observations on the omega-3 PUFA and carotenoid supplementation focused on Caucasians, and there are scarce data whether these supplements are equally associated with reduced risk of AMD in Asian populations where dietary intake of lipids are different compared to Western populations. In addition, whilst numerous studies have reviewed the association between diet and AMD condition in Western societies and in other Asian countries, there has been insufficient scientific-based report to allow validation of such an association in the Hong Kong Chinese population. Therefore, our study results could serve as a benchmark and reference for future reviews of the factors influencing AMD occurrence amongst the local Chinese.

In this current study, the role of EPA and DHA, as well as the carotenoids were consistent with the literature [16,17]. In addition, we have assessed a wide panel of omega-3 and omega-6 PUFAs, MUFAs as well as SFAs. We found that the blood level for the omega-3 PUFAs namely DHA, EPA and dihomo-γ-linolenic acid were significantly lower in AMD subjects than in the controls. In addition, a low level of DHA and EPA, as well as ALA, increased the odds ratio of developing AMD. Logistic regression also found a protective association against AMD. But for the omega-3 PUFA eicosatrienoic acid, the blood level was significantly higher in AMD, and was associated with AMD in logistic regression. ALA, EPA and DHA are attained through food sources (fish, nuts, seeds) and a good dietary habit is fundamental to maintain healthy omega-3 PUFA levels in human. The high eicosatrienoic acid and low EPA and DHA in AMD suggests a loss of Δ^5^- and Δ^6^-desaturase enzyme efficacy in the PUFA metabolism [24,25]; these enzymes are required to convert eicosatrienoic acid to EPA and DHA. For omega-6 PUFAs, the ARA and eicosadienoic acid were significantly higher in AMD compared with controls. High level of eicosadienoic acid also increased the risk of AMD, while a low ARA level was protective. Although not much is known about eicosadienoic acid, it is posed to be a metabolite of linoleic acid that may modulate the synthesis of inflammatory mediators [26]. Linoleic acid is also an essential fatty acid that is acquired from food sources such as red meat, poultry and nuts.

For MUFA, oleic acid was the most important in this study, as high-level increased the odds of AMD, and logistic regression also demonstrated its association with AMD. Moreover, for the first time, we report high SFAs levels in AMD. Most of the SFAs, except stearic acid were again associated with AMD in logistic regression, and a high blood level of these increased the odds of developing AMD. Indeed, SFAs are synthesized through de novo lipogenesis, mainly via dietary palmitic acid [27]. Our observation suggests desaturation of palmitic acid to stearic acid was rapid and eventually converted to oleic acid. It is postulated high SFA is contributed by the poor dietary habit of the AMD who had higher frequency of red meat intake, which is a rich source of SFA.

Currently, there are some studies that look at the effect of nutrient supplementation in AMD. AREDS2, which was a multi-center 5-year randomized trial designed to examine the effects of oral supplementation of macular xanthophylls (10 mg lutein and 2 mg zeaxanthin) and/or omega-3 PUFAs (EPA 650 mg and DHA 350 mg) on the progression to advanced AMD. There was no additional benefit from adding the omega-3 PUFAs or a mixture of lutein and zeaxanthin to the formulation. Although the addition of omega-3 to the AREDS formulation was not shown to be advantageous, it is believed that higher doses of EPA and DHA may have a desirable effect [3]. A small observational study reported by Georgiou and Prokopiou found dry AMD patient with severe vision loss had significant visual improvement when supplemented with EPA and DHA for up to 6 months [28]. Another recent large prospective cohort reported higher intakes of EPA and DHA (350 mg/d or fatty fish of ≥ 2 servings/week) showed moderate reduction risk of AMD and potentially be the primary prevention or method to delay the occurrence of visually significant intermediate AMD for Americans [29]. The conflicting findings in these intervention studies demonstrate that more understanding regarding the circulating levels of these nutrients are necessary. The ideal source, dosage, timing of intervention and route of omega-3 PUFA supplements were also unknown. Thus, in our current study, we focused on the actual difference in the blood omega-3 PUFA levels. To date, this was the only comprehensive case-control study that investigated the full fatty acid profiles in Chinese patients. In the Western countries, the relationship with macular pigment density and circulating levels of DHA and EPA has been studied [30]. Limpia study recently found an association between increased macular pigment optical density (MPOD) with circulating levels of omega-3 docosapentaenoic acid as well [31], but nutrient supplementation did not change the MPOD [32].

Whether the circulating levels of these micronutrients were related to the dietary habit was not well reported in the literature. However, our study confirmed the carotenoid levels were significantly lower in AMD cases, and a low blood level of these increased the odds of developing AMD. We found AMD group had significantly less intake of both green leafy vegetables as well as other plant-based food (e.g., nuts, turnip, celery, cabbage, and potatoes). These foods are rich in carotenoids, and also ALA a plant-based omega-3 PUFA where a low level of such, increased the odds ratio of having AMD. Insofar, of the omega-3 PUFA, only poor fish oil intake (EPA and DHA) were implicated in AMD but not from plants. The meat intake, including preserved meats, red meats (pork, beef and lamb), and poultry were also significantly higher in the AMD group. These foods are rich in omega-6 PUFA which are sources of pro-inflammatory eicosanoids, especially ARA, and linoleic acid, as well as the SFAs, such as palmitic acid. In our study, a high level of SFAs was found to increase the odds ratio of having AMD, and our logistic regression model also found SFAs to be associated with AMD. As for seafood, although there was no difference in the frequency intake of fish between the control and AMD, other seafood (prawn, shrimp, crab, mussels, scallop) that has lesser EPA and DHA content than fish [33], were consumed less frequently in the AMD group. Nonetheless, our study did not investigate the type of fish consumed where oily fish (mackerel, herring, salmon) contain higher omega-3 PUFA than white fish (cod, tilapia, sea bass); the latter is popular in Hong Kong Chinese diet. Nevertheless, results of the dietary analysis were not decisive because the difference in responses between cases and controls was small. This may be due in part to the subjectivity of the FFQ and therefore, the accuracy of the patient response in the questionnaire.

One of the strengths of this study was in fact the quantitative analysis of fatty acids and carotenoids of the collected blood samples from AMD subjects and age-matched controls. It has been suggested that these circulating biomarkers of lipid and the type of dietary lipid are important in the management of AMD [34]. Utilizing these data, we could examine the patterns of subjects’ dietary habits and their relationships with the aforementioned blood levels and other risk factors for AMD.

## Figures and Tables

**Table 1:** Mean difference between the mean values of each tested nutrients between age-related macular degeneration (AMD) cases and controls.

	Mean Difference between Cases & Controls	Standard Error	*p*-Value	95% Confidence	Interval
Carotenoids					
Beta-carotene	−0.050	0.024	0.036	−0.098	−0.003
Lycopene	−1.197	0.358	0.001	−1.903	−0.492
Lutein + zeaxanthin	−0.097	0.039	0.013	−0.173	−0.021
Saturated Fatty Acids (SFA)					
C14:0 (Myristic acid)	2.509	0.243	<0.0005	2.031	2.988
C15:0 (Pentadecylic acid)	2.509	0.243	<0.0005	2.031	2.988
C16:0 (Palmitic acid)	91.241	14.160	<0.0005	63.369	119.113
C17:0 (Margaric acid)	1.954	0.340	<0.0005	1.284	2.623
C18:0 (Stearic acid)	16.824	10.187	0.1	−3.229	36.876
C20:0 (Arachidic acid)	0.094	0.151	0.533	−0.202	0.390
Monounsaturated fatty acids (MUFA)					
C14:1n9 (Myristoleic acid)	0.112	0.397	0.778	−0.669	0.893
C15:1n10 (Pentadecenoic acid)	0.208	0.358	0.561	−0.496	0.912
C16:1n9 (Palmitoleic acid)	0.274	1.392	0.844	−2.465	3.013
C17:1n10 (Heptadecenoic acid)	1.511	0.589	0.011	0.351	2.670
C18:1n9 (Oleic acid)	101.543	14.282	0.0005	73.431	129.655
C20:1n9 (Gondoic acid)	0.475	0.436	0.277	−0.383	1.333
C22:1n9 (Erucic acid)	0.728	0.353	0.04	0.033	1.423
Omega-6 Polyunsaturated fatty acids (PUFA)					
C18:2n6 (Linoleic acid)	0.843	17.843	0.962	−34.279	35.964
C18:3n6 (γ-Linolenic acid)	−0.826	0.438	0.06	−1.687	0.036
C20:2n6 (Eicosadienoic acid)	1.307	0.446	0.004	0.429	2.185
C20:3n6 (Dihomo-γ-linolenic acid)	−3.906	1.230	0.002	−6.328	−1.485
C20:4n6 (Arachidonic acid)	53.703	6.834	<0.0005	40.253	67.154
C22:4n6 (Adrenic acid)	−0.226	0.350	0.519	−0.914	0.462
Omega-3 Polyunsaturated fatty acids (PUFA)					
C18:3n3 (α-Linolenic acid)	−1.899	1.122	0.092	−4.107	0.310
C20:3n3 (Eicosatrienoic acid)	8.307	1.449	<0.0005	5.456	11.159
C20:5n3 (Eicosapentaenoic acid)	−4.404	0.759	<0.0005	−5.898	−2.910
C22:5n3 (Docosapentaenoic acid)	−0.331	0.200	0.099	−0.724	0.063
C22:6n3 (Docosahexaenoic acid)	−6.651	2.134	0.002	−10.851	−2.450

**Table 2:** Plasma concentration of fatty acids in healthy control and wet AMD patients.

Concentration (μg/mL) ^†^	Healthy Control (*n* = 198)	Wet AMD (*n* = 99)
Saturated fatty acids		
C14:0 (Myristic acid)	12.03 ± 5.49	13.58 ± 3.24 *
C15:0 (Pentadecylic acid)	2.23 ± 1.40	4.67 ± 2.60 ****
C16:0 (Palmitic acid)	267.05 ± 109.79	344.98 ± 105.24 ****
C17:0 (Margaric acid)	5.02 ± 2.31	6.76 ± 2.60 ****
C18:0 (Stearic acid)	173.50 ± 81.57	190.21 ± 64.12
C20:0 (Arachidic acid)	1.03 ± 0.76	1.17 ± 0.56
Mono-unsaturated fatty acids		
C14:1n9 (Myristoleic acid)	3.20 ± 2.10	3.82 ± 1.18 *
C15:1n10 (Pentadecenoc acid)	3.68 ± 2.42	4.52 ± 1.15 **
C16:1n9 (Palmitoleic acid)	23.62 ± 9.68	24.10 ± 9.39
C17:1n10 (Heptadecenoic acid)	8.94 ± 5.14	10.40 ± 2.50 *
C18:1n9 (Oleic acid)	280.38 ± 111.20	375.69 ± 95.89 ****
C20:1n9 (Gondoic acid)	7.16 ± 3.69	7.71 ± 2.13
C22:1n9 (Erucic acid)	3.57 ± 2.15	3.57 ± 1.97
Omega-6 polyunsaturated fatty acids		
C18:2n6 (Linoleic acid)	417.46 ± 143.05	415.25 ± 81.83
C18:3n6 (γ-Linolenic acid)	3.72 ± 2.09	4.31 ± 1.96
C20:2n6 (Eicosadienoic acid)	5.48 ± 2.92	6.95 ± 3.69 ***
C20:3n6 (Dihomo−γ-linolenic acid)	17.77 ± 10.01	13.45 ± 7.81 ***
C20:4n6 (Arachidonic acid)	95.57 ± 39.81	148.84 ± 68.44 ****
C22:4n6 (Adrenic acid)	3.40 ± 2.04	2.85 ± 1.98
Omega-3 polyunsaturated fatty acids		
C18:3n3 (α-Linolenic acid)	15.02 ± 5.12	12.56 ± 3.91 ***
C20:3n3 (Eicosatrienoic acid)	17.77 ± 10.01	21.42 ± 11.66 *
C20:5n3 (Eicosapentaenoic acid)	13.10 ± 6.15	8.92 ± 4.61 ****
C22:5n3 (Docosapentaenoic acid)	2.14 ± 1.68	1.92 ± 0.99
C22:6n3 (Docosahexaenoic acid)	22.58 ± 14.95	12.80 ± 6.52 ****

AMD, age-related macular degeneration. ^†^ Values are expressed as mean ± SD. * *p* < 0.05, ** *p* < 0.01, *** *p* < 0.001, **** *p* < 0.0001.

**Table 3:** Odds ratio of being a case (AMD) in the lowest quartile, using the highest quartile as reference. Only those with a statistically significant results are shown.

	Odds-Ratio	Standard Error	*p*-Value	95% Confidence	Interval
Carotenoids					
Beta-carotene	2.779	1.091	0.009	1.287	5.997
Lycopene	6.017	2.478	<0.0005	2.685	13.488
Lutein+zeaxanthin	4.825	2.068	<0.0005	2.083	11.177
Omega-6 polyunsaturated fatty acids					
C20:4n6 (Arachidonic acid)	0.110	0.047	<0.0005	0.048	0.253
C22:4n6 (Adrenic acid)	2.594	1.025	0.016	1.196	5.630
Omega-3 polyunsaturated fatty acids					
C18:3n3 (α-Linolenic acid)	3.107	1.184	0.003	1.472	6.558
C20:3n3 (Eicosatrienoic acid)	0.160	0.075	<0.0005	0.064	0.399
C22:5n3 (Docosapentaenoic acid)	7.789	3.288	<0.0005	3.406	17.813
C22:6n3 (Docosahexaenoic acid)	6.326	2.944	<0.0005	2.541	15.750

**Table 4:** Odds ratio of being a case (AMD) in the highest quartile, using the lowest quartile as reference. Only those with a statistically significant results are shown.

	Odds-Ratio	Standard Error	*p*-value	95% Confidence	Interval
C20:2n6 (Eicosadienoic acid)	2.634	0.994	0.010	1.257	5.519
C18:1n9 (Oleic acid)	14.095	7.194	<0.0005	5.184	38.327
C16:0 (Palmitic acid)	11.484	5.640	<0.0005	4.386	30.071
C15:0 (Pentadecylic acid)	33.754	17.897	<0.0005	11.940	95.420

**Table 5:** Comparison of Food-Frequency Questionnaire (FFQ) results between healthy controls and wet AMD patients.

Food Item	Frequency of Dietary Intake Per Week *		*p*-Value	Serving Size Per Meal *		*p*-Value
	Control	AMD		Control	AMD	
Source of carotenoids						
Green leafy vegetables (e.g., Chinese kale, broccoli, spinach)	4 (1)	4 (2)	0.102	5 (1)	4 (1.5)	<0.001
Tomato	1 (7)	2 (7)	0.287	2 (2)	2 (3)	0.630
Red carrots	2 (7)	8 (7)	0.129	2 (2)	3 (2)	0.742
Other vegetables (e.g., turnip, celery cabbage, cabbage and potato)	2 (7)	2 (7)	0.284	4 (1)	3 (2)	<0.001
Source of polyunsaturated fatty acid						
Red meat (pork, beef, lamb)	2 (2)	3 (3)	0.012	3 (1)	2 (1)	0.052
Poultry (chicken, duck, pigeon)	2 (3)	2 (7)	0.031	3 (1)	2 (1)	0.030
Oily fish (mackerel, eel, woo fish, salmon, sardine)	8 (2.5)	8 (4)	0.912	3 (1)	2 (2)	0.207
White meat fish (e.g., sea bass, tilapia, cod)	3 (3)	2 (2)	0.361	3 (0)	3 (1.5)	0.204
Other seafood (e.g., prawn, seafood, crab, mussels, scallop)	7 (7)	8 (7)	0.006	2 (2)	2 (2)	0.312
Walnuts	8 (0.5)	8 (0)	0.326	1 (0)	1 (0)	0.784
Peanuts	8 (4)	8 (6)	0.733	1 (1)	1 (1)	0.671
Other nuts (e.g., almond, pistachio, cashew)	8 (6)	8 (6)	0.151	2 (1)	2 (1)	0.781
Others ^†^						
Milk	2 (1)	1 (1)	0.110			
Cheese	2 (1)	2 (1)	0.798			
Preserved vegetables	2 (1)	2 (1)	0.045			
Preserved meat	2 (1)	2 (0)	0.027			

* Data expressed in median (inter-quarter range). The statistical significance of medians between groups was measured by Mann-Whitney test (*p* < 0.05). Food items excerpted from the FFQ are related to major source of carotenoids (vegetables) and polyunsaturated fatty acids (meat, poultry and fish). ^†^ Serving size was not included in the questionnaire.

## Supplementary Materials

The following are available online at https://www.mdpi.com/2072-6643/11/8/1720/s1, Supplementary Material 1: Template for the Food-Frequency Questionnaire (FFQ) for recruited subjects, Supplementary Material 2: Methodology of plasma analysis, including detailed procedures and settings for gas chromatography-mass spectrometry and liquid chromatography with diode array detector (LC-DAD).
