# Supplementary material for: Dietary Habits, Fatty Acids and Carotenoid Levels Are Associated with Neovascular Age-Related Macular Degeneration in Chinese
Source: Nutrients. 2019 Jul 25;11(8):1720. doi: 10.3390/nu11081720 (PMC6723701; doi:10.3390/nu11081720)
Supplement: Supplementary file 1 [file nutrients-11-01720-s001.pdf]

Dietary habits, fatty acids and carotenoid levels are associated with neovascular age-related macular degeneration in Chinese

[1,2]Alex LK Ng, [3]Ho Hang Leung[†], [4]Ryo Kawasaki, [5]Wing-Lau Ho, [5]Loraine LW Chow, [5]Sharon SW Chow, [3]Jetty Chung-Yung Lee[*], [1,6]Ian YH Wong[++]

[*]Corresponding author
[†]Co-first author
[++]Co-corresponding author

1 Department of Ophthalmology, LKS Faculty of Medicine, The University of Hong Kong, Sassoon Road, Hong Kong SAR;
2 Hong Kong Ophthalmic Associate, Queen's Road, Hong Kong SAR;
3 School of Biological Sciences, The University of Hong Kong, Pokfulam Road, Hong Kong SAR;
4 Department of Vision Informatics, Osaka University, 2-2 Yamadaoka, 565-0871 Osaka, Japan;
5 Grantham Hospital, Hospital Authority, Wong Chuk Hang, Hong Kong SAR;
6 Department of Ophthalmology, Hong Kong Sanatorium and Hospital, Happy Valley, Hong Kong SAR
* Correspondence: jettylee@hku.hk; Tel.:+852 2299 0318 (JCL); +852 2572 0211 (IYW)
† The authors contributed equally to this work.
++ Co-corresponding author

Supplementary Materials 1 & 2.

Supplementary Material 1

Version no. 4, Revised on 23.9.2015

Questionnaire on the association between age-related macular degeneration and dietary habits

Date		Height	cm
Age		Weight	kg
Sex	M / F	Body Mass Index (BMI)	kg/m^2
Education Level	Primary／Secondary／Tertiary or Above ／Not Educated*	Occupation	

*Please circle the applicable option

1. Are you taking any vitamin or nutritional supplements? (e.g. fish liver oil capsules, calcium tablets)

①☐ Yes, please specify：______________________

②☐ No

2. Do you have past or present smoking habits?

①☐ Yes (please answer questions 3 and 4)

②☐ No (please answer question 5)

3. How long is your past or current smoking duration in years? ______________ years

4. How many cigarettes did or do you smoke daily in your past or current smoking habits?

____

5. How is your drinking habit now (including any kinds of alcohol, such as red wine, baijiu/white wine), brandy, whisky, rum, gin, beer, rice wine, cider and sake)?

①☐ Never drink alcohol

②☐ Once a week (please answer questions 6 and 7)

③☐ 2-3 times a week (please answer questions 6 and 7)

④☐ 4-5 times a week (please answer questions 6 and 7)

⑤☐ 6 times or above a week (please answer questions 6 and 7)

6.1 How much do you drink every time? ____________ Milliliter (mL)

6.2.What kind of alcohol do you drink? Red wine / white wine / brandy / whisky / rum / gin / beer / rice *baijiu*/ cider / sake / others, please specify: ______________

Please tick (✓) the box for appropriate answer(s)

7. Could you tell me which of the following best describes your current dietary habits?

①☐ I am a vegetarian

②☐ I am on special diet because of diabetes / renal diseases

③☐ I am on special diet (e.g, nut or gluten free) because of _________________

④☐ No special diet, I eat almost everything

⑤☐ No special diet, but I tend to eat more meats, less vegetables and fruits
⑥☐ No special diet, but I tend to eat more vegetables and fruits, and less meats

8. Please rate your preference with each of the following food?

	1<br>never eat	2<br>occasionally	3<br>often consume	4<br>always consume
**8a.** Western bakery products	☐	☐	☐	☐
**8b.** Milk	☐	☐	☐	☐
**8c.** Yogurt	☐	☐	☐	☐
**8d.** Cheese	☐	☐	☐	☐
**8e.** Tofu	☐	☐	☐	☐
**8f.** Fried tofu	☐	☐	☐	☐
**8g.** Tofu sheet	☐	☐	☐	☐
**8h.** Soy drinks	☐	☐	☐	☐
**8i.** Preserved vegetables	☐	☐	☐	☐
**8j.** Preserved meat	☐	☐	☐	☐
**8k.** Nuts	☐	☐	☐	☐
**8l.** Green tea	☐	☐	☐	☐
**8m.** Black tea	☐	☐	☐	☐

9. Could you remember the changes of your intake for the following food over the past one year?

	**1**<br>Less than before	**2**<br>Almost no change	**3**<br>Slightly more than before	**4**<br>A lot more than before
**9a.** Meat	☐	☐	☐	☐
**9b.** Seafood (e.g. fish)	☐	☐	☐	☐
**9c.** Vegetables	☐	☐	☐	☐
**9d.** Fruits	☐	☐	☐	☐

10. Could you tell me how you usually cook your food? *Maximum 2 answers for each food group.*

	**1**<br>**Steam**	**2**<br>**Stir fry**	**3**<br>**Fry**	**4**<br>**Grill**	**5**<br>**Stew**	**6**<br>**Boil**	**7**<br>**Never eat**
**10a.** Vegetables	☐	☐	☐	☐	☐	☐	☐
**10b.** Meat	☐	☐	☐	☐	☐	☐	☐
**10c.** Sea/river food	☐	☐	☐	☐	☐	☐	☐

11. Could you tell me which of the following best describe your taste preference?

①☐ Very salty ②☐ Salty
③☐ Median ④☐ Bland
⑤☐ Very bland

12. Could you tell me which of the following best describe your taste preference?

①☐ Very sweet ②☐ Sweet
③☐ Median ④☐ Bland
⑤☐ Very bland

13. Could you tell me which of the following best describe your preference of oily food?

①☐ Very oily ②☐ oily
③☐ Median ④☐ bland
⑤☐ Very bland

Interviewers READ: I am going to ask you about your diet in the past 7 days. Please think about what you ate in the past 7 days, including those in meals and as snacks.

14. Could you tell me which of the following best describe the taste of your food in the past 7 days?

①☐ Most of them are very salty ②☐ Most of them are salty
③☐ Most of them are median ④☐ Most of them are blank
⑤☐ Most of them are very blank

15a. In the past 7 days, how often did you eat rice and rice products including cooked rice, congee, rice noodle and Cheung Fan?

①☐ Never
②☐ Did not eat in the past 7 days
③☐ 1~2 / week ④☐ 3~4/ week ⑤☐ 5~6/ week
⑥☐ 1/day ⑦☐ 2/day ⑧☐ ≥ 3/ day

15b. Each time you ate rice and rice products, what is your serving size?

(1 Bowl = 100 g = 2 Liangs) *Please show subjects the sample bowl!*
①☐ < 1 bowl ②☐ 1 bowl ③☐ 1.5 bowls
④☐ 2 bowls ⑤☐ 2.5 bowls ⑥☐ ≥ 3 bowls

16a. In the past 7 days, how often did you eat wheat noodle (e.g, dan mien, ganmien, chow mien noodles, yi mien, lo mien, udon, wonton noodles, Shanghai noodles, etc)?

①☐ Never
②☐ Did not eat in the past 7 days

③☐ 1~2 / week ④☐ 3~4/ week ⑤☐ 5~6/ week
⑥☐ 1/day ⑦☐ 2/day ⑧☐ ≥ 3/ day

16b. Each time you ate wheat noodle, what is your serving size?
(1 Bowl = 100 g = 2 Liangs) *Please show subjects the sample bowl!*
①☐ < 1 bowl ②☐ 1 bowl ③☐ 1.5 bowls
④☐ 2 bowls ⑤☐ 2.5 bowls ⑥☐ ≥ 3 bowls

17a. In the past 7 days, how often did you eat cereal meal?

①☐ Never
②☐ Did not eat in the past 7 days
③☐ 1~2 / week ④☐ 3~4/ week ⑤☐ 5~6/ week
⑥☐ 1/day ⑦☐ 2/day ⑧☐ ≥ 3/ day

17b. Each time you ate cereal meal, what is your serving size?
(1 bowl = 100 g = 2 Liang)
①☐ < 1 bowl ②☐ 1 bowl ③☐ 1.5 bowls
④☐ 2 bowls ⑤☐ 2.5 bowls ⑥☐ ≥3 bowls

18a. In the past 7 days, how often did you eat Dim Sum, e.g. Man-Tau, plain roll and barbecue pork bun etc?
①☐ Never
②☐ Did not eat in the past 7 days
③☐ 1~2 / week ④☐ 3~4/ week ⑤☐ 5~6/ week
⑥☐ 1/day ⑦☐ 2/day ⑧☐ ≥ 3/ day

18b. Each time you ate Dim Sum, what is your serving size?
(1 piece of Mann-Tau/plain roll≈50g, 1 piece of barbecue pork bun≈75 g)

①☐ < 50g ②☐ 50g ③☐ 50 ~ 75g ④☐ 100 ~ 150 g ⑤☐ ≥ 200g

19a. In the past 7 days, how often did you eat bread, e.g. pineapple bun, croissant etc?
①☐ Never
②☐ Did not eat in the past 7 days
③☐ 1~2 / week ④☐ 3~4/ week ⑤☐ 5~6/ week
⑥☐ 1/day ⑦☐ 2/day ⑧☐ ≥ 3/ day

19b. Each time you ate bread, what is your serving size?
(1 piece of pineapple bun≈75g , 1 piece of bread/Croissant≈50 g)
①☐ < 50g ②☐ 50g ③☐ 50 ~ 75g ④☐ 100 ~ 150 g ⑤☐ ≥ 200g

20a. In the past 7 days, how often did you eat biscuits and cake?

① ☐ Never
② ☐ Did not eat in the past 7 days
③ ☐ 1~2 / week ④ ☐ 3~4/ week ⑤ ☐ 5~6/ week
⑥ ☐ 1/day ⑦ ☐ 2/day ⑧ ☐ ≥ 3/ day

20b. Each time you ate bread, what is your serving size?

(1 piece of Tai Ping biscuits≈10g , 1 piece of cake≈50 g)

① ☐ < 50g ② ☐ 50g ③ ☐ 50 ~ 75g ④ ☐ 100 ~ 150 g ⑤ ☐ ≥ 200g

21a. In the past 7 days, how often did you eat green leafy vegetables (Choi Sum, Pok Choi, Chinese Kale, Broccoli, spinach)?

① ☐ Never
② ☐ Did not eat in the past 7 days
③ ☐ 1~2 / week ④ ☐ 3~4/ week ⑤ ☐ 5~6/ week
⑥ ☐ 1/day ⑦ ☐ 2/day ⑧ ☐ ≥ 3/ day

21b. Each time you ate green leafy vegetables, what is your serving size?

(1 portion = 50 g = 1 Liang)

① ☐ < 1 Liang ② ☐ 1 Liang ③ ☐ 1.5 Liangs ④ ☐ 2~3 Liangs ⑤ ☐ ≥ 4 Liangs

22a. In the past 7 days, how often did you eat tomato?

① ☐ Never
② ☐ Did not eat in the past 7 days
③ ☐ 1~2 / week ④ ☐ 3~4/ week ⑤ ☐ 5~6/ week
⑥ ☐ 1/day ⑦ ☐ 2/day ⑧ ☐ ≥ 3/ day

22b. Each time you ate tomato, what is your serving size?

(1 portion = 50 g = 1 Liang)

① ☐ < 1 Liang ② ☐ 1 Liang ③ ☐ 1.5 Liangs ④ ☐ 2~3 Liangs ⑤ ☐ ≥ 4 Liangs

23a. In the past 7 days, how often did you eat red carrots?

① ☐ Never
② ☐ Did not eat in the past 7 days
③ ☐ 1~2 / week ④ ☐ 3~4/ week ⑤ ☐ 5~6/ week
⑥ ☐ 1/day ⑦ ☐ 2/day ⑧ ☐ ≥ 3/ day

23b. Each time you ate red carrots, what is your serving size?

(1 portion = 50 g = 1 Liang)

① ☐ < 1 Liang ② ☐ 1 Liang ③ ☐ 1.5 Liangs ④ ☐ 2~3 Liangs ⑤ ☐ ≥ 4 Liangs

24a. In the past 7 days, how often did you eat other vegetables? E.g. turnip, celery cabbage, cabbage and potatos.

①☐ Never

②☐ Did not eat in the past 7 days

③☐ 1~2 / week ④☐ 3~4/ week ⑤☐ 5~6/ week

⑥☐ 1/day ⑦☐ 2/day ⑧☐ ≥ 3/ day

24b. Each time you ate other vegetables, what is your serving size?

(1 portion = 50 g = 1 Liang)

①☐ < 1 Liang ②☐ 1 Liang ③☐ 1.5 Liangs ④☐ 2~3 Liangs ⑤☐ ≥ 4 Liangs

25a. In the past 7 days, how often did you eat tofu?

①☐ Never

②☐ Did not eat in the past 7 days

③☐ 1~2 / week ④☐ 3~4/ week ⑤☐ 5~6/ week

⑥☐ 1/day ⑦☐ 2/day ⑧☐ ≥ 3/ day

25b. Each time you ate tofu, what is your serving size?

(1 portion = 50 g = 1 Liang)

①☐ < 1 Liang ②☐ 1 Liang ③☐ 1.5 Liangs ④☐ 2~3 Liangs ⑤☐ ≥ 4 Liangs

26a. In the past 7 days, how often did you eat mushrooms/wood fungi?

①☐ Never

②☐ Did not eat in the past 7 days

③☐ 1~2 / week ④☐ 3~4/ week ⑤☐ 5~6/ week

⑥☐ 1/day ⑦☐ 2/day ⑧☐ ≥ 3/ day

26b. Each time you ate mushrooms/wood fungi, what is your serving size?

(1 portion = 50 g = 1 Liang)

1☐ < 1 Liang 2☐ 1 Liang 3☐ 1.5 Liangs 4☐ 2~3 Liangs 5☐ ≥ 4 Liangs

27. When you eat meat, do you remove fat/skin on the meat, or would you:

①☐ Eat all ②☐ Remove some of the fat/skin before eating

③☐ Remove all the fat/skin before eating ④☐ Do not eat fatty meat

28a. In the past 7 days, how often did you eat red meat including pork, beef and lamb?

①☐ Never

②☐ Did not eat in the past 7 days

③☐ 1~2 / week ④☐ 3~4/ week ⑤☐ 5~6/ week

⑥☐ 1/day ⑦☐ 2/day ⑧☐ ≥ 3/ day

28b. Each time you ate red meat, what is your serving size?

(1 portion = 50 g = 1 Liang)

①□ < 1 Liang ②□ 1 Liang ③□ 2 ～ 3 Liangs ④□ ≥ 4 Liangs

29a. In the past 7 days, how often did you eat poultry? This include all poultries－roasted duck, steamed/boiled chicken and stewed pigeons etc.

①□ Never

②□ Did not eat in the past 7 days

③□ 1~2 / week ④□ 3~4/ week ⑤□ 5~6/ week

⑥□ 1/day ⑦□ 2/day ⑧□ ≥ 3/ day

29b. Each time you ate poultry, what is your serving size?

(1 portion = 50 g = 1 Liang)

①□ < 1 Liang ②□ 1 Liang ③□ 2 ～ 3 Liangs ④□ ≥ 4 Liangs

30a. In the past 7 days, how often did you eat oily fish such as mackerel, eel, woo fish, salmon and sardine? DO NOT COUNT salted fish!

①□ Never

②□ Did not eat in the past 7 days

③□ 1~2 / week ④□ 3~4/ week ⑤□ 5~6/ week

⑥□ 1/day ⑦□ 2/day ⑧□ ≥ 3/ day

30b. Each time you ate oily fish, what is your serving size?

(1 portion = 50 g = 1 Liang)

①□ < 1 Liang ②□ 1 Liang ③□ 2 ～ 3 Liangs ④□ ≥ 4 Liangs

31a. In the past 7 days, how often did you eat other fish such as grass fish, big head fish, blace and golden thread fish? DO NOT COUNT salted fish!

①□ Never

②□ Did not eat in the past 7 days

③□ 1~2 / week ④□ 3~4/ week ⑤□ 5~6/ week

⑥□ 1/day ⑦□ 2/day ⑧□ ≥ 3/ day

31b. Each time you ate those fish, what is your serving size?

(1 portion = 50 g = 1 Liang)

①□ < 1 Liang ②□ 1 Liang ③□ 2 ～ 3 Liangs ④□ ≥ 4 Liangs

32a. In the past 7 days, how often did you eat other seafood such as prawn, shrimp, crab, mussels, scallop?

①□ Never

②☐ Did not eat in the past 7 days

③☐ 1~2 / week ④☐ 3~4/ week ⑤☐ 5~6/ week

⑥☐ 1/day ⑦☐ 2/day ⑧☐ ≥ 3/ day

32b. Each time you ate these seafood, what is your serving size?

(1 portion = 50 g = 1 Liang)

①☐ < 1 Liang ②☐ 1 Liang ③☐ 2 ～ 3 Liangs ④☐ ≥ 4 Liangs

33. How many eggs did you eat in past 7 days? Does this include preserved egg?

①☐ Never

②☐ < 1/ week ③☐ 1~2/ week ④☐ 3~4/ week

⑤☐ 1/day ⑥☐ 2/day ⑦☐ ≥ 3/ day

34a. In the past 7 days, how often did you eat preserved/cured meat?

①☐ Never

②☐ Did not eat in the past 7 days

③☐ 1~2 / week ④☐ 3~4/ week ⑤☐ 5~6/ week

⑥☐ 1/day ⑦☐ 2/day ⑧☐ ≥ 3/ day

34b. Each time you ate preserved/cured meat, what is your serving size?

(1 portion = 50 g = 1 Liang)

①☐ < 1 Liang ②☐ 1 Liang ③☐ 2 ～ 3 Liangs ④☐ ≥ 4 Liangs

35a. In the past 7 days, how often did you eat salted fish?

①☐ Never

②☐ Did not eat in the past 7 days

③☐ 1~2 / week ④☐ 3~4/ week ⑤☐ 5~6/ week

⑥☐ 1/day ⑦☐ 2/day ⑧☐ ≥ 3/ day

35b. Each time you ate salted fish, what is your serving size?

(1 portion = 50 g = 1 Liang)

①☐ < 1 Liang ②☐ 1 Liang ③☐ 2 ～ 3 Liangs ④☐ ≥ 4 Liangs

36a. In the past 7 days, how often did you eat pickled vegetables?

①☐ Never

②☐ Did not eat in the past 7 days

③☐ 1~2 / week ④☐ 3~4/ week ⑤☐ 5~6/ week

⑥☐ 1/day ⑦☐ 2/day ⑧☐ ≥ 3/ day

36b. Each time you ate pickled vegetables, what is your serving size?

(1 portion = 50 g = 1 Liang)

1☐ < 1 Liang 2☐ 1 Liang 3☐ 1.5 Liangs 4☐ 2~3 Liangs 5☐ ≥ 4 Liangs

37a. In the past 7 days, how often did you eat citrus fruit e.g. orange and lemon?

①☐ Never

②☐ Did not eat in the past 7 days

③☐ 1~2 / week ④☐ 3~4/ week ⑤☐ 5~6/ week

⑥☐ 1/day ⑦☐ 2/day ⑧☐ ≥ 3/ day

37b. Each time you ate citrus fruit, what is your serving size?

(1 piece of orange ≈ 3 Liangs = 150 g)

①☐ < 1 piece ②☐ 1 piece ③☐ 2 ～ 3 pieces ④☐ ≥ 4 pieces

38a. In the past 7 days, how often did you eat other fruit e.g. apple, pear and Lychee?

①☐ Never

②☐ Did not eat in the past 7 days

③☐ 1~2 / week ④☐ 3~4/ week ⑤☐ 5~6/ week

⑥☐ 1/day ⑦☐ 2/day ⑧☐ ≥ 3/ day

38b. Each time you ate these fruit, what is your serving size?

(1 portion = 50 g = 1 Liang)

①☐ < 1 Liang ②☐ 1 Liang ③☐ 2 ～ 3 Liangs ④☐ ≥ 4 Liangs

39a. In the past 7 days, how often did you eat walnuts?

①☐ Never

②☐ Did not eat in the past 7 days

③☐ 1~2 / week ④☐ 3~4/ week ⑤☐ 5~6/ week

⑥☐ 1/day ⑦☐ 2/day ⑧☐ ≥ 3/ day

39b. Each time you ate walnuts, what is your serving size?

(1 portion = 50 g = 1 Liang)

①☐ < 1 Liang ②☐ 1 ～ 2 Liangs ③☐ 3 ～ 4 Liangs ④☐ ≥ 5 Liangs

40a. In the past 7 days, how often did you eat peanuts?

①☐ Never

②☐ Did not eat in the past 7 days

③☐ 1~2 / week ④☐ 3~4/ week ⑤☐ 5~6/ week

⑥☐ 1/day ⑦☐ 2/day ⑧☐ ≥ 3/ day

40b. Each time you ate peanut, what is your serving size?

(1 portion = 50 g = 1 Liang)

①☐ < 1 Liang ②☐ 1 ～ 2 Liangs ③☐ 3 ～ 4 Liangs ④☐ ≥ 5 Liangs

41a. In the past 7 days, how often did you eat other nuts? e.g. almond, pistachio nuts and cashew etc.

①☐ Never

②☐ Did not eat in the past 7 days

③☐ 1~2 / week ④☐ 3~4/ week ⑤☐ 5~6/ week

⑥☐ 1/day ⑦☐ 2/day ⑧☐ ≥ 3/ day

41b. Each time you ate peanut, what is your serving size?

(1 portion = 50 g = 1 Liang)

①☐ < 1 Liang ②☐ 1 ～ 2 Liangs ③☐ 3 ～ 4 Liangs ④☐ ≥ 5 Liangs

42a. In the past 7 days, how often did you use soy sauce, either for cooking or as a side sauce?

①☐ Never

②☐ Did not eat in the past 7 days

③☐ 1~2 / week ④☐ 3~4/ week ⑤☐ 5~6/ week

⑥☐ 1/day ⑦☐ 2/day ⑧☐ ≥ 3/ day

42b. Could you tell me which of the following best describe the amount of your soy sauce consumption?

①☐ Very little ②☐ A little ③☐ Median ④☐ A bit more ⑤☐ Quite a lot

43a. Which type of oil/fat do you usually use in cooking?

①☐ Never

②☐ Peanut oil ③☐ Corn oil ④☐ Other vegetable oil* ⑤☐ Animal fat

***e.g, canola, olive, sunflower, rapeseed oil**

43b. Could you tell me which of the following best describe the amount of your oil consumption?

①☐ Very little ②☐ A little ③☐ Median ④☐ A bit more ⑤☐ Quite a lot

44. Could you tell me how many glasses of liquid do you usually have per day? Please count water, tea, soup, milk, soy drinks, coffee and juice etc.

(1 glass = 250 ml) *Please show subjects the sample glass!*

①☐ < 1 ②☐ 1~2 ③☐ 3~5

④☐ 6~8 ⑤☐ 8~10 ⑥☐ ≥ 10

45a. In the past 7 days, how often did you have soy drinks?

①☐ Never

②☐ Did not eat in the past 7 days

③☐ 1~2 / week ④☐ 3~4/ week ⑤☐ 5~6/ week

⑥☐ 1/day ⑦☐ 2/day ⑧☐ ≥ 3/ day

45b. Each time you had soy drinks, what is your serving size?

(1 glass = 250 ml) *Please show subjects the sample glass!*

①□ < 1 ②□ 1~2 ③□ 3~5

④□ 6~8 ⑤□ 8~10 ⑥□ ≥ 10

46a. Which type of milk do you usually consume?

①□ Never

②□ Whole milk ③□ Condensed milk ④□ Skimmed milk ⑤□ Low-fat milk

46b. In the past 7 days, how often did you drink milk?

①□ < 1/ week ②□ 1~2/ week ③□ 3~4/ week

④□ 1/day ⑤□ 2/day ⑥□ ≥ 3/ day

46c. Each time you drank milk, how many glasses did you have? DO NOT count those made from milk powder! (1 glass = 250 ml) *Please show subjects the sample glass!*

①□ < 1 ②□ 1~2 ③□ 3~5

④□ 6~8 ⑤□ 8~10 ⑥□ ≥ 10

47a. In the past 7 days, how often did you have yogurt?

①□ Never

②□ Did not eat in the past 7 days

③□ 1~2 / week ④□ 3~4/ week ⑤□ 5~6/ week

⑥□ 1/day ⑦□ 2/day ⑧□ ≥ 3/ day

47b. Each time you ate yogurt, what is your serving size?

(1 cup = 150 g) *Please show subjects the sample yogurt!*

①□ < 1 ②□ 1 ~ 2 ③□ 3 ~ 5 ④□ 6 ~ 8 ⑤□ > 8

48a. In the past 7 days, how often did you have cheese?

①□ Never → *The end*

②□ Did not eat in the past 7 days → *The end*

③□ 1~2 / week ④□ 3~4/ week ⑤□ 5~6/ week

⑥□ 1/day ⑦□ 2/day ⑧□ ≥ 3/ day

48b. Each time you ate cheese, what is your serving size?

(1 piece = 20 g)

①□ < 1

②□ 1 ~ 2

③□ 3 ~ 5

④□ 6 ~ 8

⑤□ > 8

End

Supplementary Material 2

Blood analysis

All organic solvents used were analytical grade. Ultrapure water (resistivity ≥18 M/cm) was prepared using a Milli-Q system (Millipore Co., Millford, MA, USA). Supelco® 37 component FAME mixture (No. CRM47885), nonadecanoic acid (No. 72332), methyl nonadecanoate (No. 74208), trans-lutein (No. 07168), trans-zeaxanthin (No. 14681), lycopene (No. 75051), trans-β-carotene (No. 22040) and trans-β-Apo-8`-carotenal (No. 10810) were all obtained from Sigma-Aldrich Co. (St. Louis, MO, USA).

Plasma fatty acids analysis

Plasma fatty acids were extracted and quantified using gas chromatography mass spectrometry (GC-MS) method as described by Quehenberger et al. with modification [13]. Briefly, the plasma samples were thawed on ice and centrifuged at 3000 x g for 5 min at 4°C to remove the denatured proteins. For each sample, 200 ◎L of plasma was diluted with phosphate-buffered saline solution (pH 7.4) and methanol. Nonadecanoic acid (C19:0) (1 ug/◎L in dichloromethane) was also added to the sample as an internal standard. Alkaline hydrolysis using 1M potassium hydroxide in methanol was performed to release the esterified fatty acids. The total fatty acids were collected by liquid-liquid extraction using isooctane. After the evaporation of solvent, derivatization using boron tetrafluoride was immediately carried out at 60°C. The fatty acid methyl esters formed during the derivatization process were extracted twice with hexane:diethyl ether (8:2 v/v), then dried completely at 37°C under a stream of nitrogen. The samples were re-suspended in isooctane and analysed immediately.

The derivatized samples were analysed by an Agilent 5977A mass selective detector interfaced with an Agilent 7890B gas chromatograph, equipped with an automatic sampler and a computer workstation. The injection port and GC-MS interface were kept at 240 and 250 °C, respectively. The mass spectrometer was used in the negative electrical ionization (NEI) mode. Separations were carried out on a SP®-2560 capillary column (100 m × 0.25 mm, d_f 0.20 μm, Sigma-Aldrich, USA). Helium was the carrier gas with a flow rate of 1 ml/min. The derivatized samples (1 μl) were injected into the GC injection port with a split ratio 1:100. The column temperature was maintained at 140°C for 5 min, then increased to 180°C at 8°C/min, and then raised to 210°C at 4°C/min, and further increased to 250°C at 20°C/min, then held at 250°C for 10 min. Selected ion monitoring (SIM) and full scan mode were performed to monitor the fatty acid methyl ester ions. Quantitation was achieved by relating the peak area of the individual fatty acid methyl ester with the C19:0 internal standard peak. Calibration curves

were set up over a concentration range of 2 – 400 ◎g/mL with 37 Component FAME mix. Each curve point was determined in triplicate and the regression coefficient ranged between 0.988 – 0.997.

Plasma carotenoids analysis

Carotenoids in the plasma samples were extracted and quantified using liquid chromatography with diode array detector (LC-DAD) method as described by Karppi et al. with modification [14]. (Briefly, the plasma samples were thawed on ice, in which 200 µl aliquot was transferred into an amber microcentrifuge tube and diluted with MilliQ water and ethanol-butylated hydroxytoluene (BHT, 0.01% in ethanol) containing the internal standard β-Apo-8-carotenal. Plasma carotenoids were collected by liquid-liquid extraction with hexane-BHT (0.01% BHT in hexane). The samples were then centrifuged at 1500 × g for 5 min at 4°C and frozen at −80°C to solidify the lower aqueous layer. The supernatant was transferred into a new amber auto-sampler vial, then dried under a stream of nitrogen at 37°C until complete dryness. The dried sample was reconstituted in the mobile phase, transferred to insert vials, and analysed immediately. The LC-DAD system consisting of 1260 Infinity LC system (Agilent, USA) with a C_{18} column (250 × 4.6 mm, 5.0 µm particle size, ACE, UK) maintained at 30 °C was used. The isocratic mobile phase consisted of a mixture of acetonitrile–methanol–chloroform (60:25:15, v/v/v). The flow rate was set to 1 mL/min and the injection volume was 50 µl. The total running time was 15 min. Carotenoids and internal standard β-Apo-8-carotenal were monitored at 454 nm. Quantitation was achieved by relating the peak area of the individual carotenoid with the internal standard peak. Calibration curves were set up over a concentration range of 0.01 – 10 mg/L with the standard cocktail. Each curve point was determined in triplicate and the regression coefficient ranged between 0.995 – 0.998. The carotenoids that were analyzed included beta-carotene, lycopene, and combined lutein with zeaxanthin (using the LC-DAD system, it was not possible to achieve a full separation of zeaxanthin and lutein, thus the two isomers were analyzed together).

Table 1. List of fatty acids analyzed in plasma by gas-chromatography/mass spectrometry.

**Saturated fatty acids (SFAs)**	
C14:0	Myristic acid
C15:0	Pentadecylic acid
C16:0	Palmitic acid
C17:0	Margaric acid
C18.0	Stearic acid
C20.0	Arachidic acid
**Monounsaturated fatty acids (MUFAs)**	
C14:1n9	Myristoleic acid
C15:1n10	Pentadecenoic acid
C16:1n9	Palmitoleic acid
C17:1n10	Heptadecenoic acid
C18:1n9	Oleic acid
C20:1n9	Gondoic acid
C22:1n9	Erucic acid
**Omega-6 Polyunsaturated fatty acids (PUFA)**	
C18:2n6	Linoleic acid (LA)
C18:3n6	Gamma-linolenic acid (GLA)
C20:2n6	Eicosadienoic acid (EDA)
C20:3n6	Dihomo-gamma-linolenic acid (DGLA)
C20:4n6	Arachidonic acid (AA)
C22:4n6	Adrenic acid (AdA)
**Omega-3 Polyunsaturated fatty acids (PUFA)**	
C18:3n3	Alpha-linolenic acid (ALA)
C20:3n3	Eicosatrienoic acid (ETE)
C20:5n3	Eicosapentaenoic acid (EPA)
C22:5n3	Docosapentaenoic acid (DPA)
C22:6n3	Docosahexaenoic acid (DHA)
